# Field Representation Microwave Thermography Utilizing Lossy Microwave Design Materials

**DOI:** 10.3390/s21144830

**Published:** 2021-07-15

**Authors:** Christoph Baer, Kerstin Orend, Birk Hattenhorst, Thomas Musch

**Affiliations:** Institute of Electronic Circuits, Ruhr University Bochum, Universitaetsstr. 150, 44801 Bochum, Germany; Kerstin.Orend@rub.de (K.O.); Birk.Hattenhorst@rub.de (B.H.); Thomas.Musch@est.rub.de (T.M.)

**Keywords:** microwave, thermography, field illustration, permittivity

## Abstract

In this contribution, we are investigating a technique for the representation of electromagnetic fields by recording their thermal footprints on an indicator material using a thermal camera. Fundamentals regarding the interaction of electromagnetic heating, thermodynamics, and fluid dynamics are derived which allow for a precise design of the field illustration method. The synthesis and description of high-loss dielectric materials is discussed and a technique for a simple estimation of the broadband material’s imaginary permittivity part is introduced. Finally, exemplifying investigations, comparing simulations and measurements on the fundamental TE10-mode in an X-band waveguide are presented, which prove the above introduced sensing theory.

## 1. Introduction

The measurement and visualization of electrical and magnetic fields has been an important task in the big scope of instrumentation, measurement, and education for many years as it enables a better understanding of physical phenomena. Here, several differentiations can be made for classifying methods and techniques. In terms of static and quasi-static field observation up to 100 MHz, optical sensors utilizing the Kerr effect, showing a two-dimensional field distribution, have been already reported in the 1970s [1]. Further instrumentation approaches utilize optical sensors [2,3]. However, when increasing the alternating frequency of the applied field up to wave propagation, sensor and probe design is often reduced to single point measurements that record a scalar value. Therefore, the illustration of complete field distributions is only possible by the time consuming scanning of the area of interest. Corresponding sensor and measurement techniques have been reported in [4,5,6] and include dielectric tips and dipole probes.

Anyway, the fast recording of microwave field distributions remains an interesting approach for several scientific and engineering fields including the investigation of field radiation in terms of antenna design, EMC testing and of course educational purposes. However, large scale and multi-dimensional visualization is challenging, because the desired resolution of the illustrated field image demands for a large amount of sensor array elements, which are used in near field sensor devices. Consequently, when applied to bigger investigation areas, these devices are costly, power consuming, and disturb the incident electromagnetic wave. Hence, far field measurements are often performed for antenna and electromagnetic compatibility (EMC) testing, which however give only information on radiated field components. Finally, nowadays simulation based illustrations are a key technology in antenna design and education. Yet, conformity between virtual and real world results must be verified. Another method for the illustration of electromagnetic fields and their interaction with matter is the microwave thermography (MWT) approach. Here, the thermal footprint of an incident electromagnetic wave on lossy dielectrics or metallic objects is recorded by means of a thermal camera and subsequently processed. While, materials for the illustration of two-dimensional laser beams of non-optical wavelengths, e.g., infrared lasers, are very well known and have been already commercialized and are distributed by Macken Instruments or Cascadelasers, the illustration of microwave fields is challenging because field strenghts’ are significantly lower. To date, MWT has been mostly used for non-destructive testing as described in [7]. Further recording techniques rely on thermo-elastic optical indicators [8,9], or solid state quantum sensing [10]. In [11,12,13], promising approaches for the microwave field illustration are presented that make use of films with magnetic losses in combination with thermo-fluoresecent indicators. However, due to the magnetic loss mechanism, the frequency range of the illustrated field is limited. Moreover, the presented materials demand for high feeding powers up to several watts, which result in comparably low heating and therefore low measurement effects.

Taking previous research results into consideration reveals that a frequency gap between several 100 MHz up to optical frequencies is present for field representation thermography, which we want to partly close in this paper. Next to a holistic modeling of the multi-physical background, we propose novel approaches on the description of lossy dielectric material mixing and further realizations for the accurate representation of electro-magnetic field in the microwave region between 1 GHz and 100 GHz. Obviously, for Field-Representation-MWT (FRMWT), an adequate indicator material must be found, which has to meet several requirements: its parameters regarding dielectric and thermal properties must be chosen wisely in order to increase the overall efficiency and resolution of the thermal footprint. This is desirable as it allows reduced excitation powers and the applicability of low-cost thermal cameras. Moreover, the chosen size of the indicator material plays an important role for its transparency in terms of field interference. Finally, special test benches for FRMWT must be constructed to reduce disturbing environmental influences, such as infrared reflections as well as forced and additional natural convection, which can tamper the field recording.

This manuscript is organized as follows: Section 2 explains necessary fundamentals regarding “wave-matter interaction”, “dielectric heating”, “thermal loss theory”, and “dielectric design materials and mixing”. In Section 3, we discuss and set up a novel transient FRMWT model, while Section 4 presents self-made design materials for an optimized FRMWT. Finally, Section 5 shows microwave thermography results in simulation and measurements. In this contribution, the exemplary field distribution under investigation is the fundamental TE10-mode of a rectangular X-band waveguide. Section 6 concludes the manuscript and gives an outlook on future modifications and investigations.

## 2. Fundamentals

Since the description of FRMWT demands for the combination of several physical phenomena from electromagnetics, thermodynamics, and flow dynamics, the following section gives a brief overview on the dependencies between the different disciplines.

### 2.1. Wave Interaction and Dielectric Heating

The description of the interaction between an electromagnetic wave with the indicator material is very fundamental for FRMWT. In this context we need to observe two properties: the dielectric power dissipation, and the total reflection coefficient. The dielectric power dissipation is essential for the heating process of the indicator material and therefore important for the later field illustration. The total reflection coefficient, however, is of great interest as it gives information on field interferences, which can lead to distorted field illustration. In the simplest case, a plane wave interacts with a dielectric material, which is oriented perpendicular to the Poynting-vector of the incident wave. At the interface between free space and material, reflection coefficients occur that depend on wave impedances of the free space ($Z_{0}$) and inside of the material ($Z_{1}$).

(1) $$\begin{array}{c} \Gamma =\frac{Z_{1}-Z_{0}}{Z_{1}+Z_{0}} \end{array}$$

**Figure 1:**
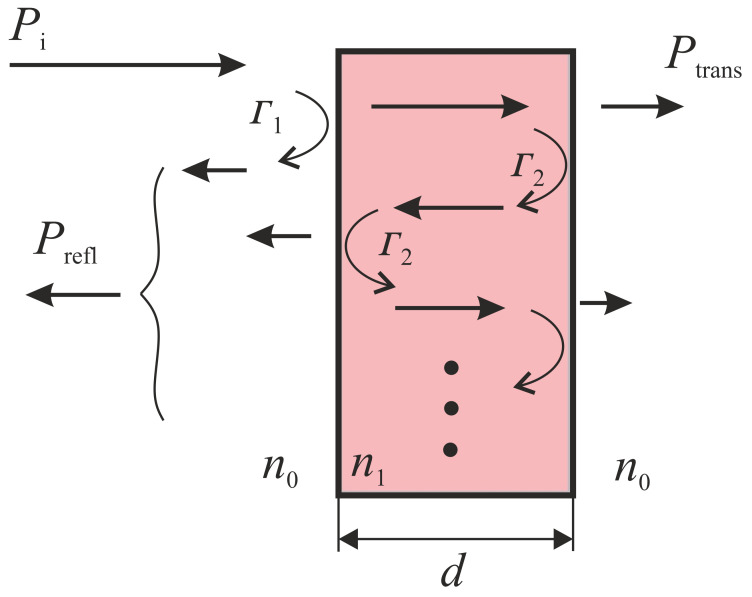
Schematical presentation of the wave interaction with a thin dielectric material sheet.

At this point, we need to distinguish between the wave’s entrance and exit reflection of the material as their coefficients differ. Furthermore, it is obvious that a part of the incident wave’s power $P_{i}$ remains inside of the material and is continuously attenuated by the material as illustrated in Figure 1. The power $P_{\mathrm{di}}$, which remains inside of the dielectric material can be calculated as follows:

(2) $$\begin{array}{c} P_{\mathrm{di}}=P_{i}\cdot \left(1-\Gamma_{1}^{2}\right)\cdot \alpha {\left(f\right)}^{2}\cdot \frac{1}{1-{\left(\Gamma_{2}\alpha \left(f\right)\right)}^{2}}. \end{array}$$

In (2), the factors $\Gamma_{1}$ and $\Gamma_{2}$ stand for the reflection coefficient at the interfaces free-space-to-material and vice versa. The attenuation α depends on the material’s thickness *d* and its dielectric properties, which are frequency dependent:

(3) α(f)=e−2πfc0Imε_r(f)·d.

The total reflection coefficient, which is important for the interference with the incident field, is the square root of the relation of the reflected power $P_{\mathrm{refl}}$ to the incident power $P_{i}$ and can be described as the superposition of the reflections at the interfaces free space-material and material-free-space. Because of the material’s thickness *d*, the contribution of the second reflection performs an additional phase shift, which must be considered for the superposition. By considering the refractive indices $n_{0}=1$ and $n_{1}=\sqrt{\varepsilon_{r},1\mu_{r},1}$ for free-space and a material, respectively. The total reflection $\Gamma_{\mathrm{tot}}$ is calculated by:

(4) $$\begin{array}{c} \Gamma_{\mathrm{tot}}=\sqrt{\frac{P_{\mathrm{refl}}}{P_{i}}}=\frac{j\left(n_{0}^{2}-n_{1}^{2}\right)\sin\left(kd\right)}{2n_{0}n_{1}\cos\left(kd\right)-j\left(n_{0}^{2}+n_{1}^{2}\right)\sin\left(kd\right)}. \end{array}$$

In (4), *j* is the imaginary unit, while *k* represents the corresponding wave number. As we consider the dielectric losses of the material, the heating process of the material is directly connected with the magnitude of the electrical field strength |E| of the incident wave. By considering the wave impedance *Z* and the length of the electric field vector $l_{1}$, it can be described by:

(5) $$\begin{array}{c} \left|E\right|=\sqrt{P_{\mathrm{di}}\cdot Z}/l_{1} \end{array}$$

Finally, the dissipated power depends on the imaginary part of the permittivity $\varepsilon_{r}^{\prime \prime }$ [14], the frequency *f* and the material’s volume *V*:

(6) $$\begin{array}{c} P_{\mathrm{loss}}=2\pi f\varepsilon_{0}\cdot \varepsilon_{r}^{\prime \prime }\cdot {\left|E\right|}^{2}\cdot V. \end{array}$$

The actual heating, which can be regarded as the power transfer from electromagnetic to thermal power, is a transient process and depends on the material’s mass *m* and its specific thermal capacity $c_{w}$. It can be described by:

(7) $$\begin{array}{c} \Delta T=\frac{P_{\mathrm{loss}}}{c_{w}\cdot m}\cdot t. \end{array}$$

Equations (5)–(7) show that the heating of an indicator material is directly proportional to the microwave excitation power. However, for calculating the electrical microwave field distribution we need to consider the square root of the recorded thermal footprint.

### 2.2. Thermal Losses

As we imbalance the thermodynamic equilibrium by the dielectric heating, the consideration of thermal losses is mandatory at this point. Otherwise, the material’s temperature would rise indefinitely, because the heating Equation (7) is a linear function with time. Consequently, different thermal loss mechanisms affect a cooling, which limits the heating process to a final temperature. Here, three main thermodynamic transfer processes must be taken into consideration: thermal conduction, radiation, and convection. In a first approximation, conduction effects can be neglected as we observe pure dielectric materials. Consequently, the main contribution to thermal losses are radiation (rad) and free convection (fc). Therefore, the total thermal power loss can be described by:

(8) $$\begin{array}{c} P_{\mathrm{th}},\mathrm{tot}=\Delta P_{\mathrm{rad}}+P_{\mathrm{fc}}. \end{array}$$

Regarding radiation losses $\Delta P_{\mathrm{rad}}$, we only need to consider the additional losses caused by the temperature difference to the surrounding. In good approximation, the phenomenon can be described using the Stefan–Boltzmann law for black bodied radiators, because for the kind of measurements carried out in this research, where temperature differences are measured, the choice of an emissivity is not fundamental. By using the Stefan–Boltzmann constant $\sigma_{\mathrm{SB}}=5.67\cdot 10^{-8}\;Wm^{-2}K^{-4}$, we receive:

(9) $$\begin{array}{cc} \Delta P_{\mathrm{rad}} & =P_{\mathrm{rad},\mathrm{Tm}-}P_{\mathrm{rad},T\infty } \end{array}$$

(10) $$\begin{array}{cc} \; & =\sigma_{\mathrm{SB}}\cdot A_{\mathrm{rad}}\cdot \left(T_{m}^{4}-T_{\infty }^{4}\right). \end{array}$$

In (9) and (10), $P_{\mathrm{rad}},\mathrm{Tm}$ and $P_{\mathrm{rad}},T\infty$ represent the thermal radiation powers caused by the temperatures $T_{m}$ and $T_{\infty }$ on the material and in the environment, respectively. The parameter $A_{\mathrm{rad}}$ indicates the heated area of the indicator material. Free convection refers to a mechanism of fluid mechanics and is caused by a spatially non-uniform distribution of density. In this case, this non-uniformity is caused by the temperature difference between indicator material and environment [15]. In a first approximation, the transported heat in steady state can be regarded as:

(11) $$\begin{array}{c} P_{\mathrm{fc}}\left(t\to \infty \right)=A_{\mathrm{rad}}\cdot \alpha_{\mathrm{th}}\cdot \Delta T_{\mathrm{env}}. \end{array}$$

In (11), $A_{\mathrm{rad}}$ is the heated area that affects free convection while $\Delta T_{\mathrm{env}}=T_{m}-T_{\infty }$ describes the temperature difference between the actual indicator material’s temperature and the environmental temperature. In case of FRMWT indicator foils, the heated area must be taken twice into consideration as free convection occurs in front and behind of the foil. The parameter $\alpha_{\mathrm{th}}$ is the heat transfer coefficient. Its determination leads to a problem of flow dynamics and therefore to the determination of the functional relationship between the non-dimensional group of Rayleigh (Ra), Prandtl (Pr), and Nusselt (Nu) numbers [16,17]. Unfortunately, this approach bases on fitted measurement models and the further investigation is not very precise. Anyway, for vertical material orientation, the literature approximates the following equations, which have been constructed from measurement series [18,19]:

(12) $$\begin{array}{c} \alpha_{\mathrm{th}}=\frac{\mathrm{Nu}\cdot \lambda_{\mathrm{th}}}{l_{1}}, \end{array}$$

(13) $$\begin{array}{c} \mathrm{Nu}={\left(0.825+0.387\cdot {\left(\mathrm{Ra}\cdot {\mathrm{Pr}}_{\mathrm{eff}}\right)}^{1/6}\right)}^{2}, \end{array}$$

(14) $$\begin{array}{c} {\mathrm{Pr}}_{\mathrm{eff}}={\left(1+{\left(\frac{0.492}{Pr}\right)}^{9/16}\right)}^{-16/9}, \end{array}$$

(15) $$\begin{array}{c} \mathrm{Ra}=\frac{g\cdot l_{2}^{3}\cdot \Delta T_{\mathrm{env}}\cdot \mathrm{Pr}}{T_{\infty }\cdot \nu^{2}}. \end{array}$$

In (12)–(15), the parameters $\lambda_{\mathrm{th}}$ and ν represent the surrounding gas’ thermal conductivity and viscosity, respectively. Further, $l_{1}$ and $l_{2}$ represent geometrical lengths for thermal imbalance alongside the direction of convection as well as the characteristic length of the flow. Finally, *g* is the gravitational acceleration. Thermal losses are highly non-linear regarding time and geometrical setup. Therefore, the presented equations must be regarded as approximation but they facilitate setting up a model for the transient behavior, which is interesting in terms of FRMWT.

### 2.3. Design Materials and Mixing

As shown before, the successful FRMWT strictly depends on the right selection of the indicator material. Here, we prefer materials that on the one hand easily heat up and store the additional temperature, while the total reflection coefficient should be kept low in order to minimize field interferences on the other hand. Moreover, thermal conduction should be kept low as well, because it would negatively affect the field illustration’s resolution. Consequently, several compromises must be met to serve these requirements. In order to meet as many requirements as possible, an indicator material was designed by mixing several raw ingredients and their corresponding physical parameters. In order to make accurate predictions, so-called mixing equations can be utilized in order to estimate final parameters. In terms of the thermal capacity $c_{w}$ and mass density ρ, the resulting quantity is directly dependent on the volume fractions ζ of the corresponding materials. In contrast to this, the description of the resulting, complex valued permittivity for dielectric mixing is not straightforward. However, the manufacturing of phantom materials for the substitution of, e.g., medical substances and surrogate explosives utilizing loss-free materials has been already published in [20,21], respectively. A general overview on dielectric mixing and corresponding models is given in [22]. In terms of FRMWT-materials, the most relevant material parameter are the losses in microwave range. Losses in dielectric materials can be caused by a small remaining conductivity, which leads to small conduction currents, or by replacement currents, which describe dipole orientation etc. Usually, for the general description of a material’s complex valued permittivity those effects are tantamount. However, referring to the Wiedemann–Franz law [23], electrical and thermal conductivity are directly proportional so that an increased electrical conductivity will deteriorate the resolution of the FRMWT. Consequently, it is important to tune the material’s losses by increasing replacement currents but keeping conductivity as low as possible. Since the electrical polarization strongly depends on the time-variation of the excitation, the permittivity depends on the frequency of those field variations. This effect is called dispersion and can be seen in both real and imaginary part of the permittivity. Part of a material’s dispersion is caused by the different polarization effects like orientation polarization as well as atomic and electronic polarization, which cause a resonance behavior in the permittivity’s real part associated with a peak in the imaginary part [22]. In any real material, several polarization mechanisms take place and superimpose, which contribute to different dispersion effects within the entire electromagnetic spectrum. Obviously, a holistic model is difficult to formulate. That is why in the literature several polarization response models can be found that describe the aforementioned dispersion and loss effects for limited frequency ranges. Because the proposed FRMWT approach in this manuscript handles the microwave frequency range, we choose the Debye model henceforth. The Debye model is an easy to understand model that considers single relaxations processes very well. As we only handle solid materials in this work, we do not expect various relaxation processes in the microwave region, but continuously decreasing permittivity’s real part values. Therefore, the Debye model is a good choice for modeling our indicator materials. Because of the induced torque of the dipole moments, the polarization requires time to reach equilibrium. This relaxation time τ is essential for the description of the complex valued permittivity. Moreover, Debye model defines the edges of the observed spectrum. Therefore, $\varepsilon_{r},\infty$ and $\varepsilon_{r},s$ describe the permittivities at optical and low frequencies, respectively. The description of a mixed material with Debye-type inclusions in a dispersion and loss-free background with permittivity $\varepsilon_{r},e$ can be aligned to the basic Maxwell–Garnett mixing rule. The resulting material is also of Debye-type and can be formulated as follows:

(16) $$\begin{array}{c} \varepsilon_{r,\mathrm{eff}}=\varepsilon_{r,\infty ,\mathrm{eff}}+\frac{\varepsilon_{r,s,\mathrm{eff}}-\varepsilon_{r,\infty ,\mathrm{eff}}}{1+j\omega \tau_{\mathrm{eff}}}. \end{array}$$

Consequently, the parameters of the mixed material are:

(17) $$\begin{array}{c} \varepsilon_{r,\infty ,\mathrm{eff}}=\varepsilon_{r,e}+\frac{3\zeta \varepsilon_{r,e}\left(\varepsilon_{r,\infty -}\varepsilon_{r,e}\right)}{\varepsilon_{r,\infty +2}\varepsilon_{r,e-\zeta }\left(\varepsilon_{r},\infty -\varepsilon_{r,e}\right)}, \end{array}$$

(18) $$\begin{array}{c} \varepsilon_{r,s,\mathrm{eff}}=\varepsilon_{r,e}+\frac{3\zeta \varepsilon_{r,e}\left(\varepsilon_{r,s}-\varepsilon_{r,e}\right)}{\varepsilon_{r,s}+2\varepsilon_{r,e}-\zeta \left(\varepsilon_{r,s}-\varepsilon_{r,e}\right)}, \end{array}$$

(19) $$\begin{array}{c} \tau_{\mathrm{eff}}=\tau \frac{\left(1-\zeta \right)\varepsilon_{r,\infty }+\left(2+\zeta \right)\varepsilon_{e}}{\left(1-\zeta \right)\varepsilon_{r,s}+\left(2+\zeta \right)\varepsilon_{e}}. \end{array}$$

Next to the Maxwell–Garnett averaging of the static and optical permittivity in (17) and (18), respectively, (19) reveals that the relaxation time of the including particles is also altered [22]. This is of great interest for the FRMWT as the material mixing allows for an optimization of the losses for a predefined operation frequency.

**Figure 2:**
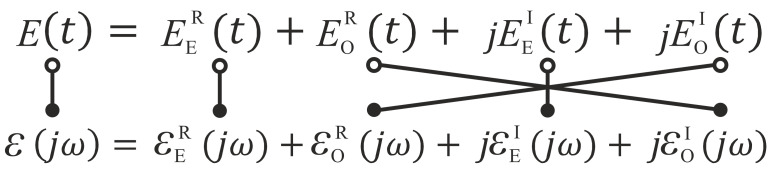
Dependencies of complex-valued time signal and the corresponding spectrum sub-divided into even (E) and odd (O) valued functions. The handle bar shows the intercorrespondencies.

Since the measurement of lossy materials results in extremely low signal-to-noise ratios, especially the determination of the the loss-describing part, i.e., the imaginary part of the permittivity, can be crucial. Moreover, measurement probes are prone to delivering wrong loss values when the probe-material connection is not properly realized and generates radiation. However, obviously the connection between frequency behavior of the real and imaginary part can be formulated. Starting from the basic physical principle of causality, which means that the polarization response cannot lead the cause, the Kramers–Kronig relation must be fulfilled for the real and imaginary part of the permittivity [24]. As consequence, this very fundamental connection allows for an estimation of the imaginary part when the broadband real part permittivity is known. The estimation itself can be done in various ways, such as solving the Kramers–Kronig integrals, applying Hilbert transformation [25], or simply using the complex valued completion (CVC) which is described further on. The CVC allows for the estimation of the permittivity’s imaginary part, when the corresponding permittivity’s real part is known. It makes use of the dependencies of complex valued time signals and their spectra, which are subdivided in their even and odd signal and spectral parts. Figure 2 illustrates the well known dependencies. Within the figure, the sign 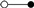 indicates the Fourier transformation from time (∘) to frequency domain (•). Moreover, the indices E and O represent even and odd signal and spectral parts, while the superscripts R and I indicate real and imaginary parts. As initial point, we consider a measured broadband, real part permittivity. As this quantity depends on frequency, we can regard it as a spectrum. Moreover, any commercial measuring probe will deliver measurement values for positive frequencies only. Therefore, the measured permittivity is the combination of its even and odd part:

(20) $$\begin{array}{c} \varepsilon_{r}^{R}=1/2\left(\varepsilon_{r},E^{R}+\varepsilon_{r},O^{R}\right). \end{array}$$

**Figure 3:**
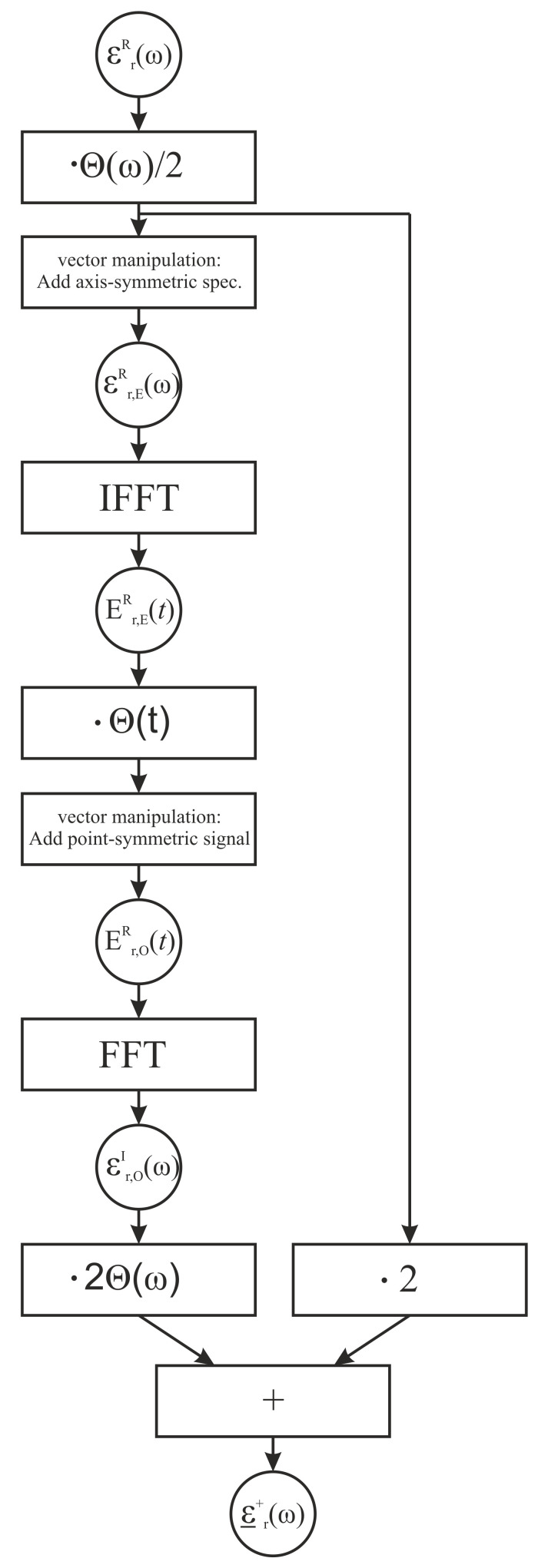
Processing steps of the complex valued completion (CVC). While rectangular boxes describe signal operation, circles indicate the current signal form. The applied function Θ indicates the heaviside function in frequency-domain (ω) and time-domain (*t*).

By means of the following CVC-steps, illustrated in Figure 3, we can calculate the right-sided, complex permittivity. As initial step we form the evenly-shaped spectrum of the measured real part permittivity. Therefore, the spectrum $\varepsilon_{r}^{R}$ is multiplied by the heaviside step function Θ and divided by 2. Additionally, the resulting data vector is mirrored to the negative frequency range in order to achieve an axis-symmetric spectrum, which is described as the evenly-shaped spectral part of the real part permittivity $\varepsilon_{r,E}^{R}$. According to Figure 2, the application of the inverse Fourier transformation delivers the evenly-shaped impulse response of the real part permittivity $E_{r,E}^{R}\left(t\right)$. In [22], several interpretations of this pulse response signal are given, however in our case the time domain signal is a pure mathematical intermediate step and further interpretation is not relevant. As the next step, we transform the evenly-shaped impulse response to be oddly-shaped. Therefore, we once more apply the heaviside function and add the point symmetric signal for t<0. By applying the Fourier transformation to the oddly-shaped time signal, we receive the oddly-shaped spectrum of the permittivity’s imaginary part $\varepsilon_{r},O^{I}\left(\omega \right)$. By doubling this spectrum, applying another heaviside step function, and adding the right-sided spectrum of the real part permittivity, we finally receive the right sided complex valued permittivity ${\underline{\varepsilon }}_{r}^{+}\left(\omega \right)$.

It needs to be mentioned that the CVC is only applicable if the measured permittivity data is sufficiently broadband and covers all relevant polarization behaviors. In terms of FRMWT and the corresponding design material manufacturing, CVC plays an important role for the precise description of the high loss indicator materials as well as their corresponding mixing equations.

## 3. Transient FRMWT Model

**Figure 4:**
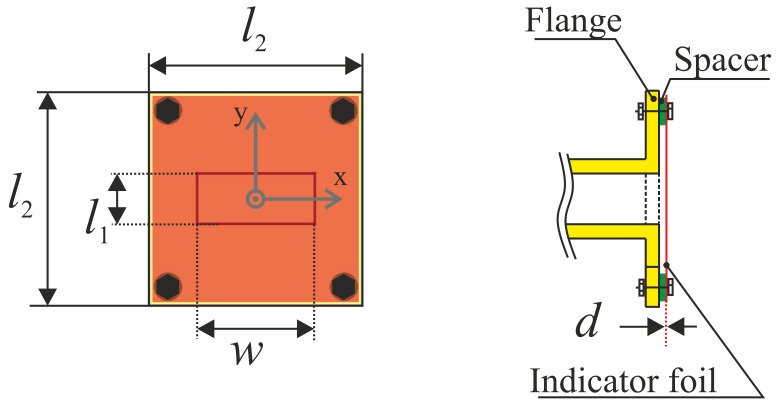
Sketch of the investigated waveguide flange.

In the following, we will observe the special case of FRMWT for mode characterization of a rectangular waveguide in X-band, as illustrated in Figure 4. The corresponding geometrical dimensions for the investigated WR90 rectangular waveguide and IEC 60154-2:2016 flange are: $l_{1}=10.16$ mm, $l_{2}=41.4$ mm, and w=22.86 mm. Moreover, the indicator material’s thickness is considered to be d=0.9 mm and it is positioned in close proximity to the waveguide’s flange.

**Figure 5:**
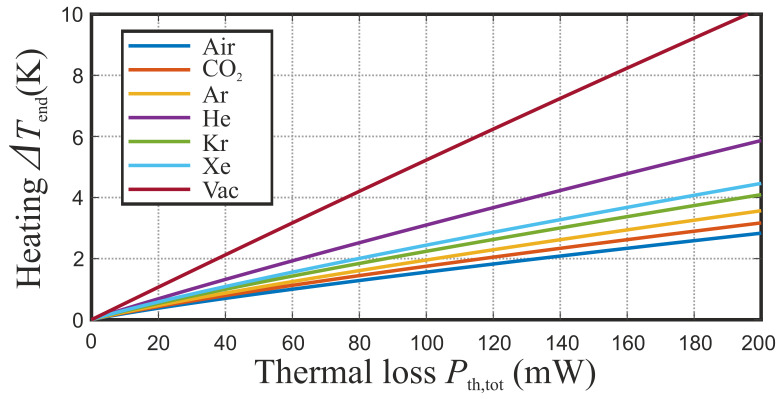
Theoretical temperature change for maximum thermal losses in steady state and different gaseous environments.

However, it does not touch its metal boundary in order to prevent additional thermal conduction. After reaching steady state, i.e., when the microwave power which is transferred to heat equals the thermal losses, the heating-up process stops and the indicator material’s end-temperature $T_{\mathrm{end}}$ is reached. Consequently, by equating the dissipated microwave power with the thermal loss power, we can calculate $T_{\mathrm{end}}$ and therefore the final temperature change $\Delta T_{\mathrm{end}}$. Figure 5 combines the thermal heating model with the microwave scenario by showing the theoretical temperature change $\Delta T_{\mathrm{end}}$ as a function of the thermal loss power for the described setup and different surrounding gases. The determination of the temperature change is of great interest as it has direct influence on the sensitivity of the thermal sensor and the applied microwave power. Therefore, for a given thermal sensor sensitivity, the required microwave power can be reduced, which is beneficial in many scenarios. Apparently, a higher temperature change is reached when choosing surrounding gases like Helium or Xenon. However, the highest temperature change is achieved when the setup is kept in vacuum, because free convection vanishes and only thermal radiation contributes to the thermal loss. Anyway, this insight shows that the construction of an optimized FRMWT test stand that guarantees for a highly defined environment scenario is desirable. Yet, low cost setups also yield sufficient results in many applications.

Since the heating process of the indicator material can be interpreted as charging process, the transient description of microwave induced heating in FRMWT can be expressed as:

(21) $$\begin{array}{c} \Delta T\left(t\right)=\Delta T_{\mathrm{end}}\cdot \left(1-e^{-\frac{\beta_{\mathrm{th}}}{\Delta T_{\mathrm{end}}}\cdot \frac{P_{\mathrm{loss}}}{c_{w}\cdot m}\cdot t}\right)=\Delta T_{\mathrm{end}}\cdot \left(1-e^{-\frac{t}{\tau_{\mathrm{th}}}}\right). \end{array}$$

In (21), the parameter $\beta_{\mathrm{th}}$ is called thermal retardation, a form factor that compensates measurement setup geometries etc. Once the thermal retardation is evaluated the time constant $\tau_{\mathrm{th}}$ can be obtained. It combines all the previous theoretical considerations from electromagnetics in Section 2.1 and thermodynamics in Section 2.2. This time constant is of great interest, because it gives indication for the compromise that needs to be found for FRMWT-measurements: On the one hand, the induced temperature differences of the indicator should be as high as possible in order to meet the thermal resolution of the utilized recording device, such as a thermal camera. On the other hand, the footprint recording should be performed as fast as possible after initial excitation in order to prevent image blurring and further non-linear behavior such as conduction effects. Therefore, we propose to take the thermal snapshot for field illustration purposes within the linear part of the transient model described in (21), i.e., for

(22) $$\begin{array}{c} \tau_{\mathrm{th}}<t_{\mathrm{rec}}<3\cdot \tau_{\mathrm{th}}, \end{array}$$

So that the heating process already reached between 68% and 95% of the final temperature.

## 4. Design Materials

**Figure 6:**
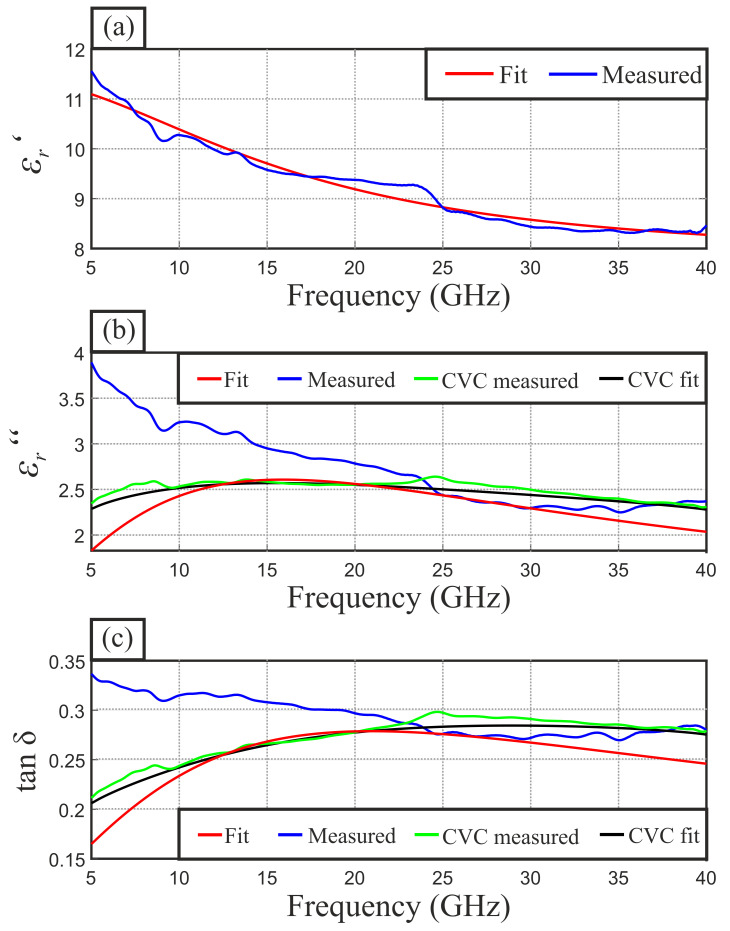
Exemplary results of the mixed material investigation at an carbon black volume fraction of $\zeta_{\mathrm{cb}}=0.07$, indicating the real (**a**) and imaginary part (**b**) permittivity as well as the loss tangent (**c**) for (—) measured values, (—) Debye model fitted values, (—) CVC of measured values and (—) CVC of Debye model fitted values.

For the manufacturing of adequate indicator material foils, several material mixtures have been investigated. In order to meet the materials requirements, i.e., providing high dielectric losses by keeping the conductivity low and enabling the possibility of forming robust foils, we chose epoxy resin as matrix material and carbon black as additive. The utilized epoxy resin Breddepox E300 from Breddemann is a low viscosity, two component casting resin, providing a high UV resistance. As carbon black, we used the DUREX 0-Powder from manufacturer Orioncarbons. This amorphous carbon black provides low conductivity, offers very good processability, exhibits low compression set, and has good dynamic properties by keeping high elasticity. For tuning and fitting the mixing equation, we produced several test mixtures with different carbon black volume fractions $\zeta_{\mathrm{cb}}$ between 0.01 and 0.09, which than were characterized by means of a DAK1.2E dielectric probe kit from SPEAG. As mentioned earlier, the measurement of high loss materials can lead to inaccuracies, especially for the permittivity’s imaginary part. Figure 6a–c show the broadband measured real and imaginary part as well as the loss tangent, respectively, for a volume fraction of $\zeta_{\mathrm{CB}}=0.07$. In (a) we added a fitting curve, which is the result of a least-square-fit of the Debye models permittivity from (16). While in (a) this curve perfectly balances the measured values, it absolutely mismatches the measured imaginary part in (b). Therefore, we added CVC-processed curves from both measurement and fitting data. Obviously, as the fitting curves and CVC-processed curve are nearly identical, the directly measured imaginary part is highly erroneous and will be ignored further on.

**Figure 7:**
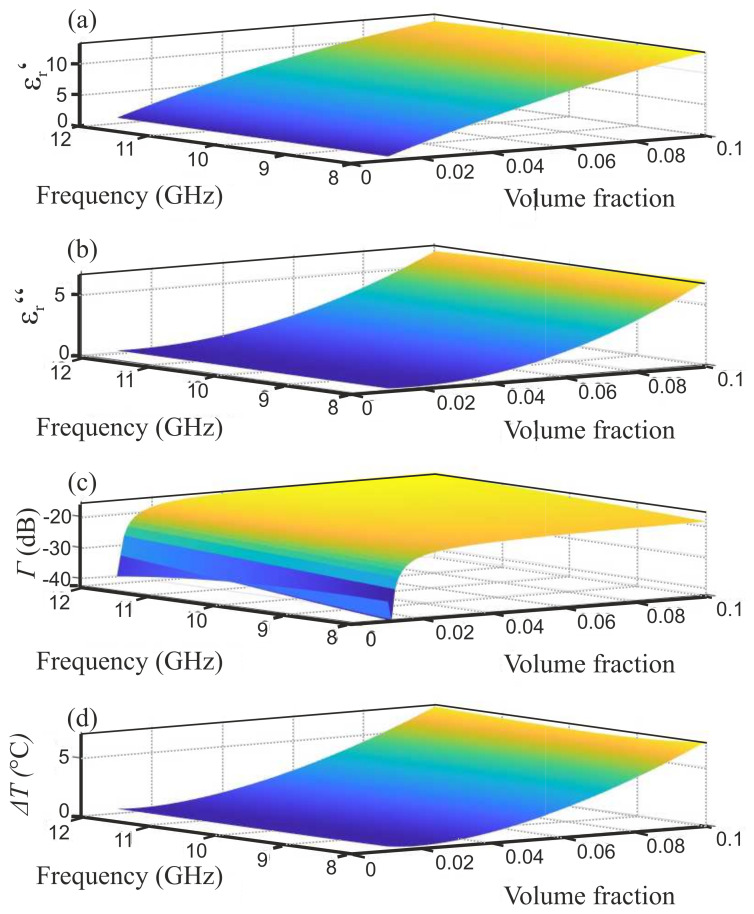
Resulting parameters of the fitted material mixing equation showing (**a**) real part permittivity, (**b**) imaginary permittivity, (**c**) total reflection coefficient, (**d**) maximum heating. Subfigures (**c**,**d**) apply for the above described indicator material and measurement scenario.

Consequently, we used the direct-fitted data for setting up the mixing equations. Figure 7 shows several 3D plots indicating the properties of processable indicator materials such as the complex permittivity (a) + (b), total reflection coefficient (c), and the expected, maximum heating (d) for the scenario described above. In the following investigation, we are using an indicator material foil with a volume fraction of $\zeta_{\mathrm{CB}}=0.05$, which allows for homogeneous mixing and a temperature increase of more than 2 ${}^{\circ }$C at 10 GHz when applying 500 mW microwave power.

## 5. Results

In order to investigate the applicability of FRMWT and to validate the presented models, several simulations and measurements have been performed on a simple test scenario. The chosen scenario is the fundamental TE10-mode within a WR90 rectangular waveguide utilizing a IEC 60154-2:2016 flange as introduced in Figure 4. The indicator material was selected from the considerations made in Section 4 and provides a complex permittivity of ${\underline{\varepsilon }}_{r}=7.2+j1.6$; as operating frequency we chose 10 GHz and an excitation power of 500 mW in both, simulations and measurements. As the waveguide will operate in fundamental mode, we expect a cos-shaped E-field distribution along side the x-axis of the waveguide. However, since the square of the electrical field strength contributes the thermal heating as shown in (6) we expect a cos${}^{2}$-shaped heat distribution. It needs to be mentioned that because the waveguide is truncated it will excite evanescent modes as well, which will slightly interfere with the fundamental mode resulting in minimal distortion of the thermal foot print.

### 5.1. Measurement Setup

From theoretical approximations in Section 2.2 and Section 3, we can conclude that the observable temperature increase for FRMWT applications will be only several kelvin, when applying reasonable microwave power. Therefore, we need to build a measurement environment, which prevents thermal and environmental disturbances. In this work, the thermal footprint of the device under test will be recorded by means of a high resolution thermal camera. Usually, these kind of cameras are portable so that it can be integrated into a measurement chamber. Further key properties of this chamber are:

- Housing:
  The measurement chamber needs to be closed and air tight for enabling different measurement environments such as gases. Moreover, it must prevent forced convection.
- Utilized Materials:
  The housing should be impervious for infrared radiation for preventing disturbing environmental reflections. The metal content should be kept low or needs a microwave absorber covering in order to avoid disturbing reflections.
- Peripheral Connections:
  Gas in- and outlets allow for FRMWT in different surrounding gases. Microwave connectors are necessary for feeding the structure under test.

**Figure 8:**
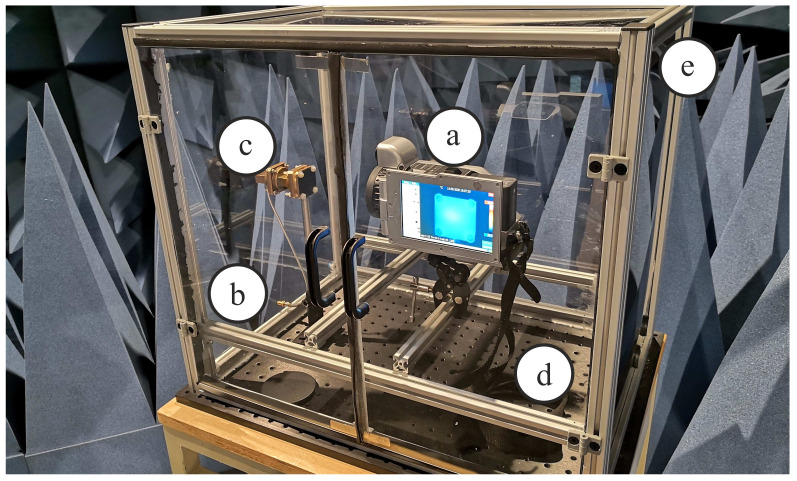
Photography of the realized setup containing: (**a**) Thermal camera, (**b**) through-hole microwave connection, (**c**) microwave device under test, (**d**) universal mechanical mounting board, (**e**) rear absorber wall.

The constructed measurement chamber was built of Plexiglas plates as these sufficiently attenuate infrared propagation within the spectrum of interest between 3 and 15 µm. The chamber measures a total size of (height, width, length) 50cm×55cm×45cm. Gas in- and outlets can be used for flooding the whole setup with gases, while an SMA-through-hole connector allows for the connection of microwave devices within a frequency range of 0.1–30 GHz. Furthermore, an optical breadboard as lower wall allows for an universal connection of mechanical components. Figure 8 shows a photography of the described measurement chamber. As thermal camera we used a VarioCAM HD research 900 from Infratec. This thermal camera records a spectral range from 7.5 to 14µm by means of an uncooled microbolometer focal plane array, which results in an image resolution of 2048×1536 pixels and a thermal resolution of 20 mK. Because the indicator material was manufactured from epoxy resin as matrix material we used an emissivity of 0.96 and a spectral range of 8 µm to 14 µm for all recordings, which refers to the values recommended by the thermal camera manufacturer [26]. The influence of further parameters is neglected in this work, because we mainly focus on temperature differences and its evolution over time.

### 5.2. Simulation and Measurement Results

In order to investigate the applicability of the indicator material the FRMWT-concept, multi-physics simulations as well as measurements with a high definition thermal camera were performed and compared. For all simulations we used the simulation software CST Microwave Studio 2020, which allows for the coupling of three-dimensional full wave analysis with a thermal simulation of the corresponding loss distributions. In terms of the microwave investigation the time domain solver with a simulation accuracy of −30 dB was utilized. In order to ensure accurate simulation results, a hexahedral mesh type was used with a mesh of approximately 1.2 million meshcells. In a subsequent step, thermal simulations were carried out, basing on the microwave simulation results in order to track the heating behavior over time and to illustrate the temperature distribution caused by the microwave losses.

**Figure 9:**
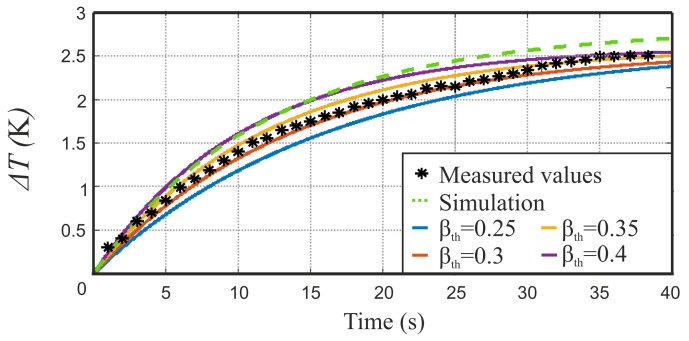
Transient temperature observation of the investigated waveguide’s center, indicating the FRMWT model for different model parameters (solids), simulation results (– –) and measurement results (*).

**Figure 10:**
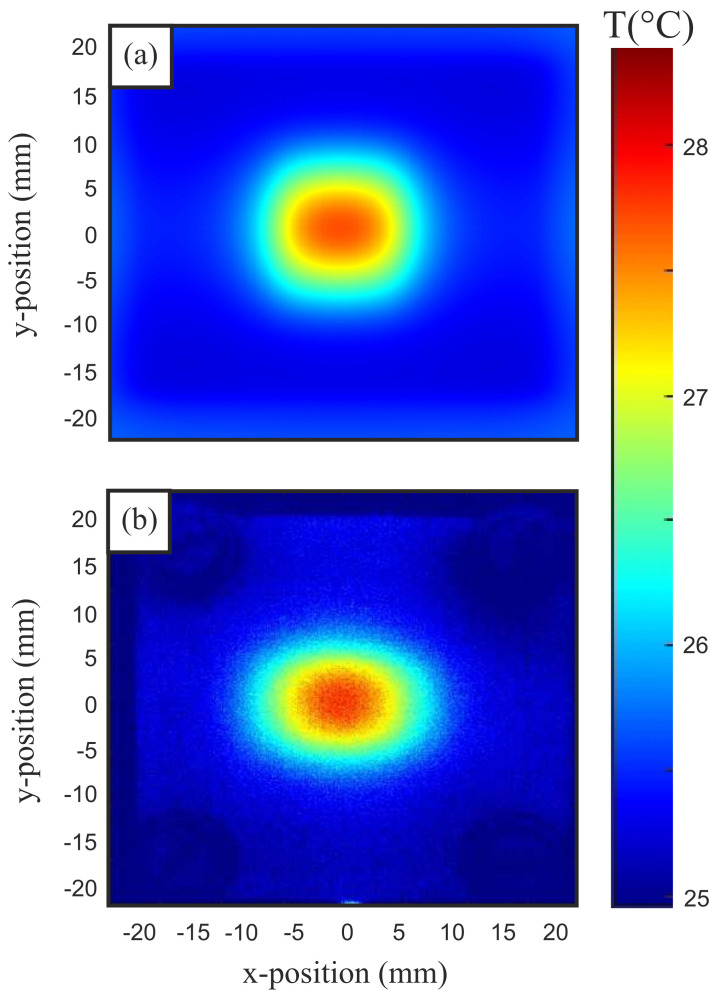
Thermography images of the investigated waveguide from (**a**) simulation and (**b**) thermal camera.

For this purpose, the convective heat transfer coefficient $\alpha_{\mathrm{th}}$ was set to 15.3 ${\mathrm{Wm}}^{-2}\;K^{-1}$. Due to the gap between material and waveguide, convection was assumed on both sides. Thermal conduction was not considered further due to the experimental setup. In addition, the material parameters of the indicator foil were included. These contain the previously measured permittivity ${\underline{\varepsilon }}_{r}=7.2+j1.6$. Further material parameters were calculated from the raw materials’ datasheets in order to agree with the measurement setup. Here, we set the heat capacity to be $c_{w}=894\;J\;{\left(\mathrm{kg}\;K\right)}^{-1}$, the mass density to be $\rho =1100\;\mathrm{kg}\;m^{-3}$ and the material thickness to d=0.9 mm. The remaining geometrical dimensions apply as described above. All simulations were carried out with an microwave excitation power of 500 mW. The following thermography images were captured on a truncated WR90 rectangular waveguide surrounded by an IEC 60154-2:2016 flange. The test recording was performed at an operating frequency of 10 GHz and a power of 500 mW, for keeping the measurement results comparable to the simulation. For the verification of the proposed transient model from Equation (21), we recorded the setup’s hotspot temperature over time in both simulation and measurement. By subtracting the initial temperature, the temperature increase over time is obtained, which is presented in Figure 9 and compared to the transient FRMWT model from (21) for different thermal retardation values $\beta_{\mathrm{th}}$. The steady state temperature difference was determined to be 2.5 K and 2.71 K in simulation and measurement, respectively. The discrepancy between simulation and measurement is 0.21 K, which is an acceptable, relative difference of 7.7% and can be explained by a different implementation of the thermal losses within the simulation tool. Because of the slightly different final temperature, the transient behavior also slightly differs between simulation and measurement as presented in Figure 9. However, the measurement fits very good to the transient FRMWT model from (21) when choosing the thermal retardation to $\beta_{\mathrm{th}}=0.35$. After the determination of all relevant parameters, the thermal time constant is calculated to be $\tau_{\mathrm{th}}=12$ s. Consequently, the following thermal images are recorded at $t_{\mathrm{rec}}=35$ s, which perfectly satisfies Equation (22). The recorded heat distributions, resulting from simulation and measurement, are shown in Figure 10a,b, respectively. In both images, the hotspot is located in the waveguide’s center position as expected because of the E-field maximum of the fundamental mode. Towards the edges of the material the heating decreases as expected. However, in y-direction the expected rectangular shape is flattened due to a remaining thermal conductivity and fringe fields in the flange-material-interspace.

**Figure 11:**
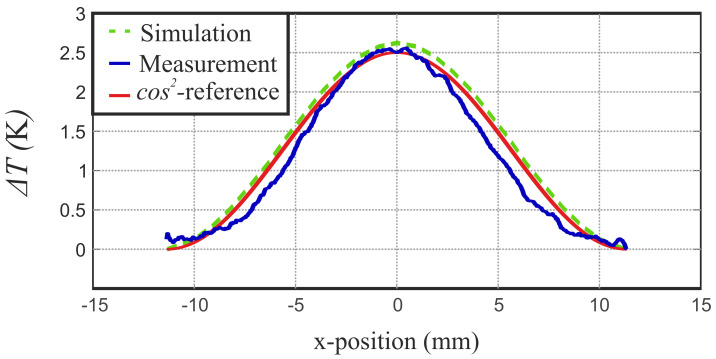
Temperature distribution of the heating in the waveguide’s center alongside the x-axis for simulation (– –) and measurements (–) compared to the theoretical $cos^{2}$-shape (–).

Figure 11 shows the temperature distributions alongside the waveguide’s x-axis in comparison to a normalized cos${}^{2}$ function. Both, simulation and measurement results, clearly reveal the expected field distribution. However, the simulation result fit the cos${}^{2}$-reference a little better. An explanation for this could be the combination of evanescent fields at the truncated waveguide flange as well as the neglected thermal conduction in simulation.

## 6. Discussion and Conclusions

In this contribution, we present a method called Field Representation Microwave Thermography (FRMWT) for the fast and accurate recording of electromagnetic fields in the microwave range. Theoretical background from microwave engineering, thermodynamics, and fluid mechanics has been derived in order set up a holistic theory, which allows the prediction of the desired field illustration. Regarding the necessary indicator material, the so called complex valued completion (CVC) has been introduced, which allows for the measurement validation of high loss materials. Investigations on mixed high-loss materials showed the necessity of the CVC for dielectrics, as straight forward measurements delivered erroneous values for the material permittivity’s imaginary part. Further, within simulations and practical experiments, we demonstrated the applicability of FRMWT using the example of the fundamental TE10-mode of rectangular waveguides. Within these investigations, we proved both, the theoretical transient FRMWT model as well as the ability of the field illustration by recording the predicted $cos^{2}$-shape of the TE10 mode’s thermal foot print. Compared to previous work, which already showed the applicability of field representation in low frequency ranges up to 100 MHz and in the optical region, this work accesses the gap-region of micro- and mmWaves from 1 GHz up to 100 GHz. Although the proposed theoretical FRMWT model makes use of several approximations, it provides an excellent and holistic foundation for future measurement setups. The introduced lossy material modeling and complex valued completion combines previous work on dielectric material mixing and gives easy access to future material modeling. Of course, its accuracy is limited to the preciseness of a priori material-knowledge and the accuracy of the necessary broad material characterization. The presented measurement setup for the FRMWT already showed a good functionality. However, as we still make use of an extremely expensive thermal camera, the current setup is only useful for laboratory measurements. In order to establish the FRMWT in practical fields, the thermal camera must be substituted. In future work, further investigations on more complex propagation modes at higher frequencies will be performed and a post-processing concept will be evaluated, which delivers key facts on the investigated mode such as the mode purity. 

## Data Availability

Not applicable.

## Abbreviations

The following abbreviations are used in this manuscript:

FRMWT	Field Representation Microwave Thermography
CVC	Complex Valued Completion
